# The role of loops B and C in determining the potentiation of GABA_A_ receptors by midazolam

**DOI:** 10.1002/prp2.433

**Published:** 2018-11-13

**Authors:** Olivia A. Moody, Andrew Jenkins

**Affiliations:** ^1^ Neuroscience Program Graduate Division of Biological and Biomedical Sciences Laney Graduate School Emory University Atlanta Georgia; ^2^ Departments of Anesthesiology & Pharmacology Emory University Atlanta Georgia

**Keywords:** allosteric modulator, benzodiazepine, canonical loops, GABA(A) receptor

## Abstract

Many benzodiazepines are positive allosteric modulators (PAMs) of GABA_A_ receptors that cause sedation, hypnosis, and anxiolysis. Benzodiazepines bind GABA_A_ receptors at the extracellular interface of the α and γ subunits. Within the α subunit, the benzodiazepine binding site is defined by three highly conserved structural loops, loops A‐C. Although previous mutagenesis studies have identified His102 in Loop A as important for benzodiazepine modulation of GABA_A_ receptors, the functional roles of many of the other conserved residues in loops A‐C remain incompletely understood. In this study, we made single mutations in loops A‐C of the benzodiazepine binding‐site across all six α subunits. We used whole‐cell patch clamp recording to measure the functional effects of these mutations on midazolam potentiation. The results showed that mutating the threonine in loop B and serine in loop C (Thr163 and S206 in human α1) did not abolish the receptors’ responsiveness to midazolam, as the α1(H102R) mutation did. The loop C mutations exhibited a novel array of α‐isoform specific effects on midazolam potentiation. The α3(S230I) and α5(S209I) mutations had the largest effect on midazolam potentiation, increasing the efficacy of midazolam. Novel benzodiazepines targeting loop C may represent a future direction for designing new drugs that specifically alter the activity of α3‐ and α5‐containing GABA_A_ receptors.

**Abbreviations**
cDNA: complementary DNA
EC_50_: 50% effective concentration
EGTA: ethylene glycol‐bis (β‐aminoethyl ether)
GABA: γ‐aminobutyric acid
GABA_A_: γ‐aminobutyric acid type A
GFP: green fluorescent protein
HEK293T: human embryonic kidney cells expressing the SV40 T‐antigen
HEPES: N‐2‐hydroxyethylpiperazine‐N‐2‐ethanesulfonic acid N, N, N’, N’‐tetra acetic acid
PAM: positive allosteric modulator

## 1 INTRODUCTION

**Figure 1:**
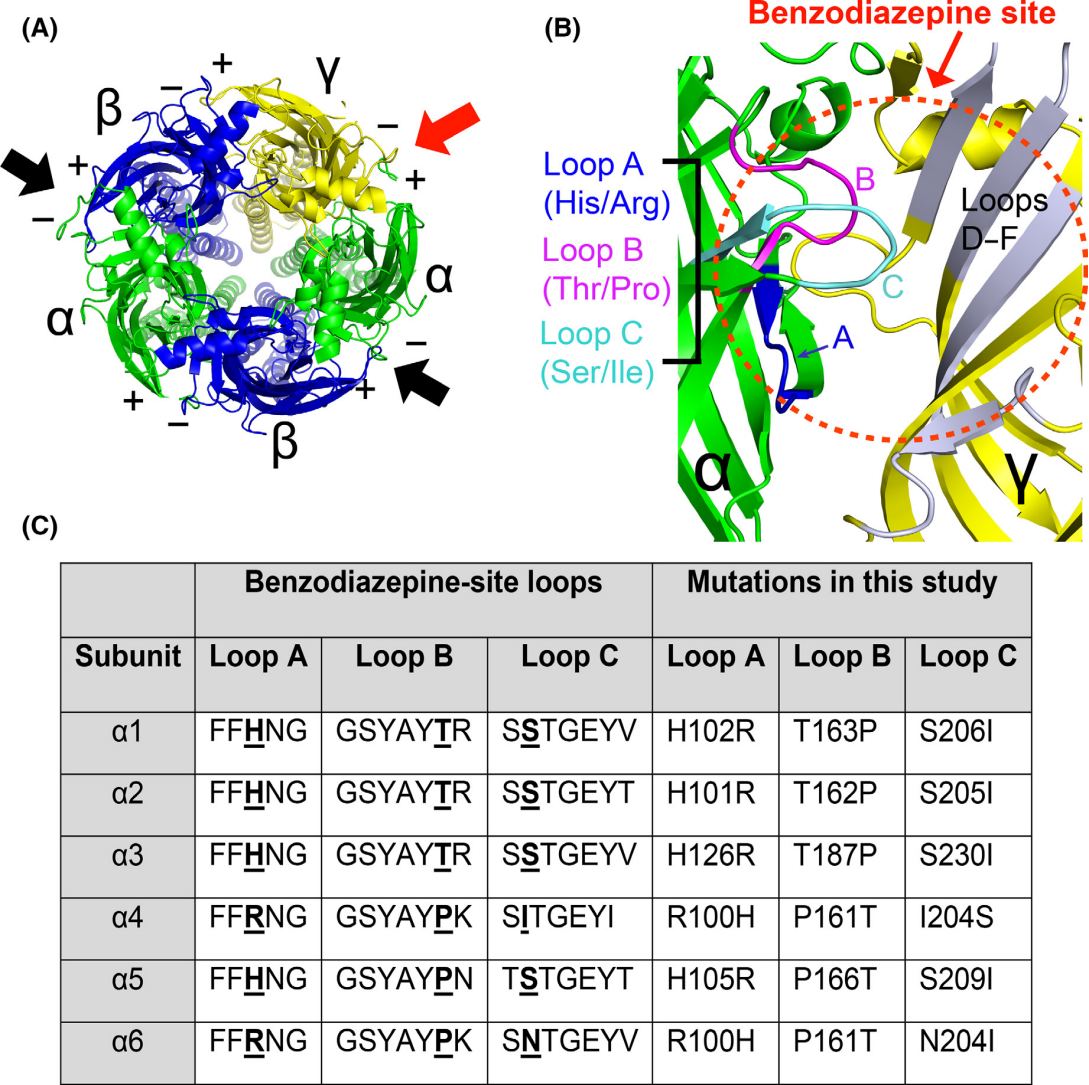
The structural loops A‐C within the α subunit form the benzodiazepine binding site on the GABA_A_ receptor. (A) The assembly of the α_x_β_2_γ_2_ GABA_A_ receptor with arrows pointing to the two GABA sites (black) and high‐affinity benzodiazepine site (red). (B) The structural loops A‐C (blue, magenta, cyan) on the α subunit and loops D‐F (grey) on the γ subunit form the benzodiazepine site (red dotted circle) on the α_x_β_2_γ_2_ receptor. Target residues used in this study noted under loops. (C) The structural loops A‐C are highly conserved across GABA_A_ receptor α subunits. The location of the residues of interest are highlighted in **bold** with the specific mutation numbers listed to the right. The numbering is based on the human mature peptide sequences not including the signal peptide (peptide sequences based on NP_000797 (α1), NP_000798 (α2), NP_000799 (α3), NP_000800 (α4), NP_000801 (α5), NP_000802 (α6)). The mutations made in this study are referred to by the abbreviations “loop A”, “loop B” and “loop C” in subsequent figures and text

Benzodiazepines can induce sedation, anxiolysis, amnesia, seizure reduction, and muscle relaxation by enhancing inhibitory GABAergic neurotransmission through the γ‐aminobutyric acid type A (GABA_A_) receptors.^1^, ^2^ GABA_A_ receptors are cys loop ligand‐gated ion channels assembled from five subunits (α1‐6, β1‐3, γ1‐3, δ, ε, θ, π, ρ1‐3) around a central pore.^3^ GABA_A_ receptors have a stoichiometry of two α subunits, two β subunits and one auxiliary subunit (predominantly γ or δ) (Figure 1A). Each subunit has a different spatial, temporal and pharmacological profile in the brain.^4^, ^5^ Upon GABA binding, the receptor's anion channel opens, causing hyperpolarizing membrane potentials in the adult mammalian brain. One highly relevant class of positive allosteric modulators (PAMs) of GABA_A_ receptors are benzodiazepines.

Benzodiazepines bind at the extracellular interface of the α and γ subunits.^6^ There are three structural loops (loops A, B, and C) on the α‐subunit and three loops on the γ2 subunit (loops D, E, and F) that form the structure of the benzodiazepine binding site (Figure 1B). Loops A‐C form connectors between sequential β‐strands. They are sometimes referred to as loop 5 (loop A), loop 8 (loop B) and β‐sheet 10 (loop C), based on nomenclature for the acetylcholine‐binding protein.^7^, ^8^ Loops A‐C are highly conserved across GABA_A_ receptor subunits and form a homologous GABA agonist binding site at the β+/α‐ interface.^9^, ^10^

A combination of mutagenesis with functional or binding assays has been used to determine the role of specific amino acids within the structural loops A‐F of the benzodiazepine site.^11^, ^12^, ^13^, ^14^, ^15^, ^16^ The conserved histidine in loop A (His101 in rodents and His102 in bovine and human cDNAs) is important for the molecular and behavioral actions of diazepam using in vitro experiments^6^, ^11^ and knock‐in mice.^2^, ^17^ Other residues in loops A‐C have been studied, but most mutagenesis experiments were constrained to mutating less than three α subunit isoforms. This limits the conclusions drawn. Many benzodiazepine ligands bind to multiple GABA_A_ receptor assemblies, and a mutagenesis study across the six α subunits is needed to determine the structural role of specific residues on benzodiazepine efficacy and potency.

In this study, we examined two residues within the conserved loops B and C across all six α subunits. The conserved threonine in loop B (GSYAY**T**R) and serine in loop C (S**S**TGEYV) have been reported to differentially affect the potency and efficacy of benzodiazepine‐site ligands, including that of zolpidem, eszopiclone, flumazenil, and β‐carbolines.^12^, ^18^, ^19^, ^20^, ^21^ It is less understood how these specific residues affect the functional actions of nonspecific positive benzodiazepines across the six human α subunits. In this study, we mutated the highly conserved histidine in loop A (His102 in α1), threonine in loop B (Thr163 in α1), and serine in loop C (Ser206 in α1) in all six GABA_A_ α subunits. The α4 and α6 subunits have different residues (R100, P161, and I/N204) in these locations (Figure 1C) and form GABA_A_ receptors insensitive to classic benzodiazepines, historically known as diazepam‐insensitive receptors.^22^ If midazolam acts as a canonical benzodiazepine then canonical mutations in α1‐3 and α5 to residues present in α4 and α6 should block its actions and *vice versa* in α4 and α6. Whole‐cell patch clamp recording was used to measure the actions of midazolam on mutated α_x_β_2_γ_2s_ GABA_A_ receptors. Midazolam was selected for this study because it is commonly used in the clinic to induce sedation,^23^ it is easier to handle than other benzodiazepines (lower affinity for diazepam‐sensitive receptors and higher solubility), and knowledge of its pharmacology could provide insight into designing novel sedatives with fewer side effects. We found that mutating the threonine and serine in loop B and loop C altered the efficacy of midazolam less than mutating the histidine in loop A across α1‐6. Surprisingly, mutating the serine in loop C altered the efficacy of midazolam potentiation in different directions depending on the α isoform. These subunit‐selective observations will be useful for the design of α3‐ and α5‐selective benzodiazepines.

## 2 MATERIALS AND METHODS

### 2.1 cDNA plasmids and mutagenesis

Human (*Homo sapiens*) GABA_A_ subunits (α1‐6, β2, γ2s) were subcloned into pcDNA3.1+ vectors with a cytomegalovirus (CMV) promoter. The hβ2 and hα3 sequences were humanized rat (*Rattus norvegicus*) cDNA with amino acid substitutions made to match the human protein sequence. The α1‐3, α5, β2, and γ2s subunits were a generous gift from Neil L. Harrison (Columbia University Medical Center, NY). The α4 subunit was obtained from GenScript (Piscataway, NJ), and the α6 subunit was a generous gift from Robert L. McDonald (Vanderbilt University, TN). All point mutations (listed in Figure 1C) were introduced using the QuikChange Lightening site‐directed mutagenesis kit (Agilent Technologies, Santa Clara, CA) according to the manufacturer's instructions and were confirmed by sequencing (Eurofins MWG Operon, Louisville, KY).

### 2.2 Cell culture and transfection

Human embryonic kidney cells containing the SV40 T‐antigen (HEK293T) were acquired from American Type Culture Collection (ATCC^®^, Manassas, VA), catalogue number, CRL3216. HEK293T cells were maintained at 37°C and 5% CO_2_ in Eagle Minimum Essential Medium (MEM) supplemented with 5% fetal bovine serum (Atlanta Biologicals Inc., Flowery Branch, GA), 40 μM l‐glutamine, 100 U/mL penicillin and 0.1 mM streptomycin. Cells were passaged regularly when they reached 70% confluency using trypsin. Cells were not passaged more than 22 times. New cells were revived from frozen stocks at passage 2‐4. Cells used for in vitro electrophysiology experiments were grown on poly‐D‐lysine‐coated glass coverslips (No.2, VWR, Radnor, PA) and transfected with X‐tremeGENE (Roche Diagnostics, Indianapolis, IN) with the desired receptor subunit cDNAs at a 1:1:1 ratio to express α_x_β_2_γ_2s_ receptors (2 μg total cDNA) and with 0.5 μg green fluorescent protein (GFP) as an expression marker. The γ2s incorporation into receptors was tested with zinc inhibition assays regularly.^24^ Patch clamp experiments were performed on cells at 24‐72 hours post‐transfection. All experiments were performed at 22°C. Experiments consisted of at least five cells recorded per day from at least two transfections across 3‐4 days to control for cell health and transfection efficiency. At least three cells expressing wild‐type receptors were recorded on days that mutant receptors were tested to provide a time‐matched expression control. All reagents were purchased from Sigma‐Aldrich (St. Louis, MO) unless otherwise stated.

### 2.3 In vitro electrophysiology

Wildtype and mutant GABA_A_ receptors were characterized using whole‐cell voltage‐clamp electrophysiology of HEK293T cells expressing α_x_β_2_γ_2s_ receptors and GFP, similar to methods previously described.^25^ Patch pipettes were created from thin‐walled borosilicate glass (TW150F‐4, World Precision Instruments, Inc., Sarasota, FL) using a horizontal puller (P97, Sutter Instruments, Inc., Novato, CA) to give a resistance of 2‐8 MΩ when filled with intracellular solution (120 mM KCl, 2 mM MgCl_2_, 10 mM EGTA, 10 mM HEPES, and adjusted to pH 7.2 with NaOH, 315 mOsm). Extracellular solution contained 161 mM NaCl, 3 mM KCl, 1 mM MgCl_2_, 1.5 mM CaCl_2_, 10 mM HEPES, and 6 mM D‐glucose, adjusted to pH 7.4 with NaOH (320‐330 mOsm). GABA and midazolam (Hospira, Lake Forest, IL) were delivered using a rapid solution changer (RSC‐160, BioLogics Science Instruments, Seyssinet‐Pariset, France) connected to a 10‐channel infusion pump (KD Scientific Inc., Holliston, MA). The perfusion system was controlled by protocols written in pClamp 9 (Molecular Devices, LLC., Sunnyvale, CA). Whole‐cell currents were recorded at ‐60 mV, filtered at 100 Hz and sampled at 200 Hz with a MultiClamp 700B amplifier (Molecular Devices, LLC) and DigiData 1322A (Molecular Devices, LLC) digitizer. *GABA concentration‐response assays* were performed by exposing each whole‐cell patch to eight concentrations of GABA spread over a 3.5 logarithmic decade. Each GABA exposure was for 2 seconds with 8 seconds of washout between ligand application. GABA concentrations for α_x_β_2_γ_2_ receptors were: 0.3‐1000 μM (α1), 0.1‐300 μM (α2 & α3), 0.03‐100 μM (α4), and 0.01‐30 μM (α5 & α6). *Midazolam concentration‐response* assays were performed by exposing patches to two successive EC_10_ (10% effective concentration) GABA exposures and then exposing the patches to ascending concentrations of coapplied midazolam (10, 50, 100, 500, 1000 nM) and GABA (EC_10_) (See Figure S1). Each midazolam drug exposure consisted of 3 seconds of coapplied GABA + midazolam and then 2 seconds of GABA at the end of each midazolam exposure before 5 seconds of washout in extracellular solution (see Figure S1 for waveform of drug exposure). GABA pre‐ and postcontrol runs were performed before and after each midazolam assay for each cell to verify a consistent EC_10_ GABA response and full washout of midazolam. Control runs consisted of 3 seconds of GABA (EC_10_) and then 3 seconds of a saturating GABA concentration (100‐300 μM depending on the α subunit) with 8 seconds of washout between ligand applications. Cells were recorded with the midazolam protocol no more than two times to avoid desensitization and incomplete washout or irreversible modulation.

### 2.4 Whole‐cell analysis

Recordings were analyzed using MATLAB (Math Works, Inc., Natick, MA). *GABA concentration‐response relationship*: Whole‐cell peak currents (*I*) were measured from GABA concentration‐response assays and fit using a nonlinear regression analysis based on the Hill equation: *I = I*_max_ *[*A*]^nH^/(EC_50_^nH^ *+ *[*A*]^nH^) where *I* was the peak current amplitude, *I*_max_ was maximum current amplitude, EC_50_ was the half‐maximal GABA concentration, *A* was the agonist concentration, and nH was the Hill coefficient. The maximum peak current, EC_50_ and Hill coefficient were estimated for assays from each cell. When Hill parameters are estimated from whole‐cell recordings, the changes in the parameters across receptor conditions can be ascribed to the following changes in receptor physiology. Changes in maximum current can be due to changes in the single‐channel conductance or the rate of desensitization. Changes in cell surface receptor expression can cause minor changes in maximum current, but are unlikely to occur in the time course of our experimental protocol. Changes in the Hill coefficient can be due to changes in altered GABA cooperativity, the loss of a GABA binding site or altered channel desensitization. More often minor changes in the Hill coefficient are attributed to the altered homogeneity in the receptor population expressed by the HEK293T cell. For example, a shallower Hill coefficient could be caused by a shift in the population of receptors from mostly αβγ receptors to a combination of both αβ and αβγ receptors. Changes in GABA apparent‐affinity can be due changes in GABA's binding affinity, gating or both for the receptor. Other explanations than the above are possible but less likely. *Midazolam concentration‐response curves*: The midazolam potentiation (%) of each GABA‐evoked response was calculated by the equation: *Pot*_**_ *= *(*I*_MDZ_ *– I*_*G*_)/*I*_*G*_ x 100%, where *Pot* was potentiation (%), *I*_*G*_ was the average amplitude of peak currents from the two EC_10_ GABA responses, and *I*_MDZ_ was the amplitude of peak currents from co‐applied GABA + midazolam. The potentiation measurements from midazolam concentration‐response curves were fit using the Hill equation: *P = P*_max**_ *[*M*]^nH^ */*(EC_50_^nH^_* *_+_* *_[*M*]^nH^)*,* where *P* was potentiation, *P*_max_ was maximum potentiation, EC_50_ was the midazolam concentration producing the half‐maximal potentiation response*, M* was midazolam concentration, and nH was the Hill coefficient. Concentration‐response relationships that were not described by a sigmoidal function were not included in our analysis (eg: no response or a linear nonsaturating response). The Hill equation was fit to each individual cell's concentration‐response curve data.

### 2.5 Statistics

Optimal sample sizes (n ≥ 10 cells) were calculated beforehand from preliminary α1 mutant data using G*Power (Heinrich‐Heine‐Universität Düsseldor, Germany) (*α* = 0.05 and *β* = 0.8) for a one‐way analysis of variance (ANOVA) test. Hill parameters (maximum response or potentiation, Hill coefficient, EC_50_) from concentration‐response curves (GABA and midazolam each) were compared for significant differences within each α subunit (α1‐6) and its loops A‐C mutants using a one‐way ANOVA at the significance threshold of *α* = 0.05. Where the results of the ANOVA were significant (*P *<* *0.05), Dunnett's post‐hoc analysis for multiple comparisons (α = 0.05) was performed. Statistical analysis was carried out using Prism 7.0 (Graphpad Software, Inc., La Jolla, CA).

## 3 RESULTS

We hypothesized that mutating single residues in the conserved loops A‐C of the benzodiazepine binding site (Figure 1C) would alter the modulation of GABA_A_ receptors by midazolam. Whole‐cell patch clamp recording of α1‐6‐containing α_x_β_2_γ_2s_ GABA_A_ receptors was used to measure the degree of potentiation by midazolam within the therapeutically relevant range of 10‐1000 nM.^26^, ^27^, ^28^ Midazolam potentiation was measured as the percent of enhancement in GABA‐evoked currents. A 100% potentiation was a doubling in amplitude of the whole‐cell current relative to the control EC_10_ GABA‐response. Interestingly, we found that loop C mutations in α3 and α5 GABA_A_ subunits increased the maximum potentiation by midazolam. However, single residue mutations in loop B and loop C did not alter, abolish or confer midazolam sensitivity as dramatically as the histidine‐to‐arginine exchanges in loop A.

### 3.1 Loop A mutations

**Figure 2:**
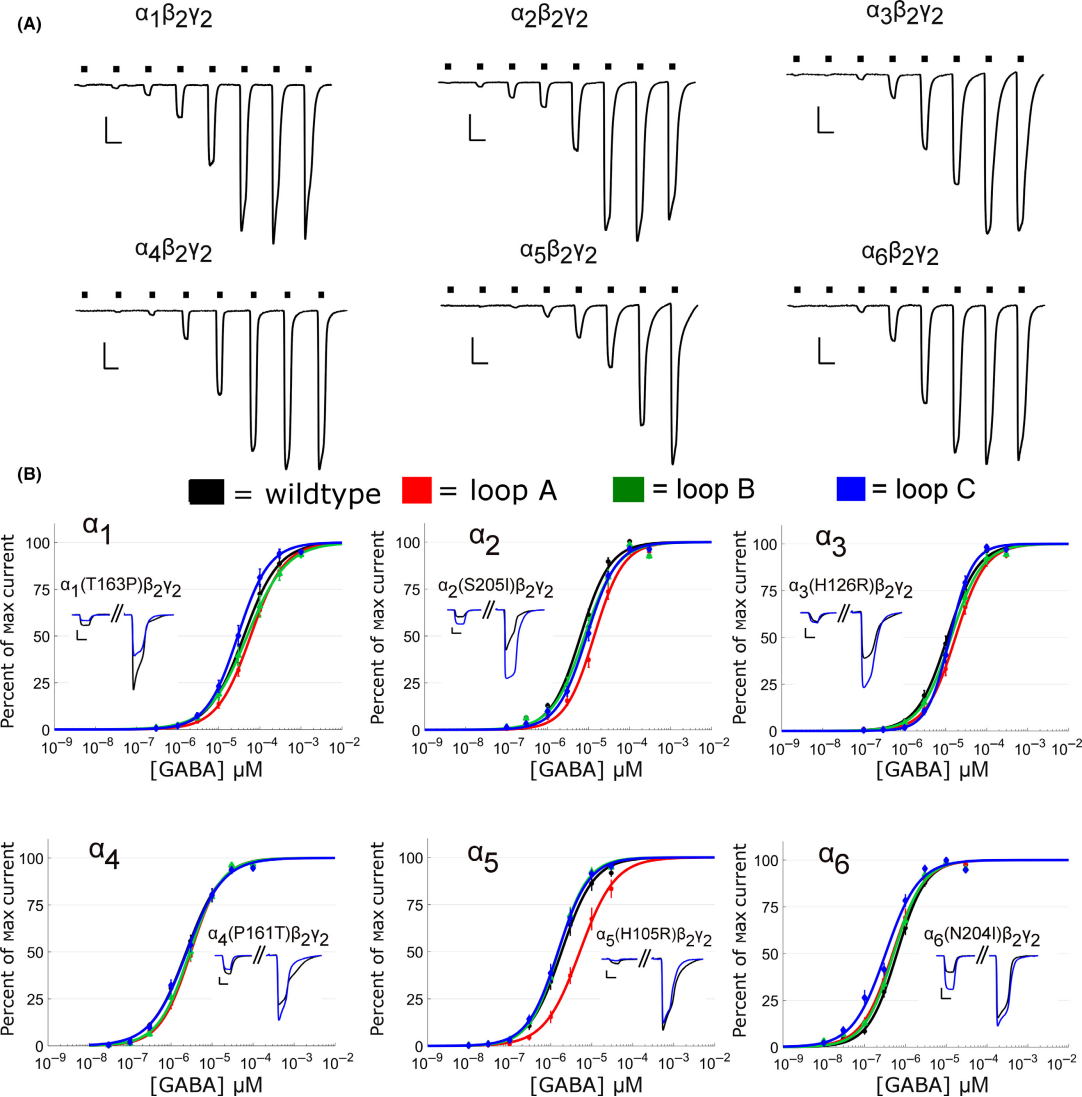
Mutations in loops A‐C across the α1‐6 subunits generally had only subtle effects on the GABA concentration‐response curves. (A) Example traces for wildtype α1‐6‐containing receptors were measured using whole‐cell patch clamp recording of HEK293T cells expressing α_x_β_2_γ_2_ receptors. GABA concentrations (black bars) were: α1 = 0.3‐1000 μM, α2 & α3 = 0.1‐300 μM, α4 = 0.03‐100 μM, and α5 & α6 = 0.01–30 μM. Scale bars: 5 seconds, 500 pA. (B) GABA concentration‐response curves of wildtype *vs* mutated receptors for each of the α subunits. Line colors: wildtype (black), loop A mutation (red), loop B mutation (green), loop C mutation (blue). Loop A mutations are α1(H102R), α2(H101R), α3(H126R), α4(R100H), α5(H105R), and α6(R100H). Loop B mutations are α1(T163P), α2(T162P), α3(T187P), α4(P161T), α5(P166T), α6(P161T). Loop C mutations are α1(S206I), α2(S205I), α3(S230I), α4(I204S), α5(S209I), and α6(N204I). Insets within each subplot are example responses from the 4th and 8th GABA concentration exposures measured for wildtype receptors (black) and one selected loop mutation (in blue). Subplot GABA concentrations: α1 (10 μM and 1000 μM), α2 (3 μM and 300 μM), α3 (3 μM and 300 μM), α4 (1 μM and 100 μM), α5 (0.3 μM and 30 μM), α6 (0.3 μM and 30 μM). Scale bars are 5 sec, 500 pA. Sample sizes (cells per group) are: α1 (10), α2 (9‐40), α3 (11‐16), α4 (12‐14), α5 (10‐12), and α6 (7‐15). Points are mean ± SEM and where SEM is smaller than symbols, it is not visible

**Figure 3:**
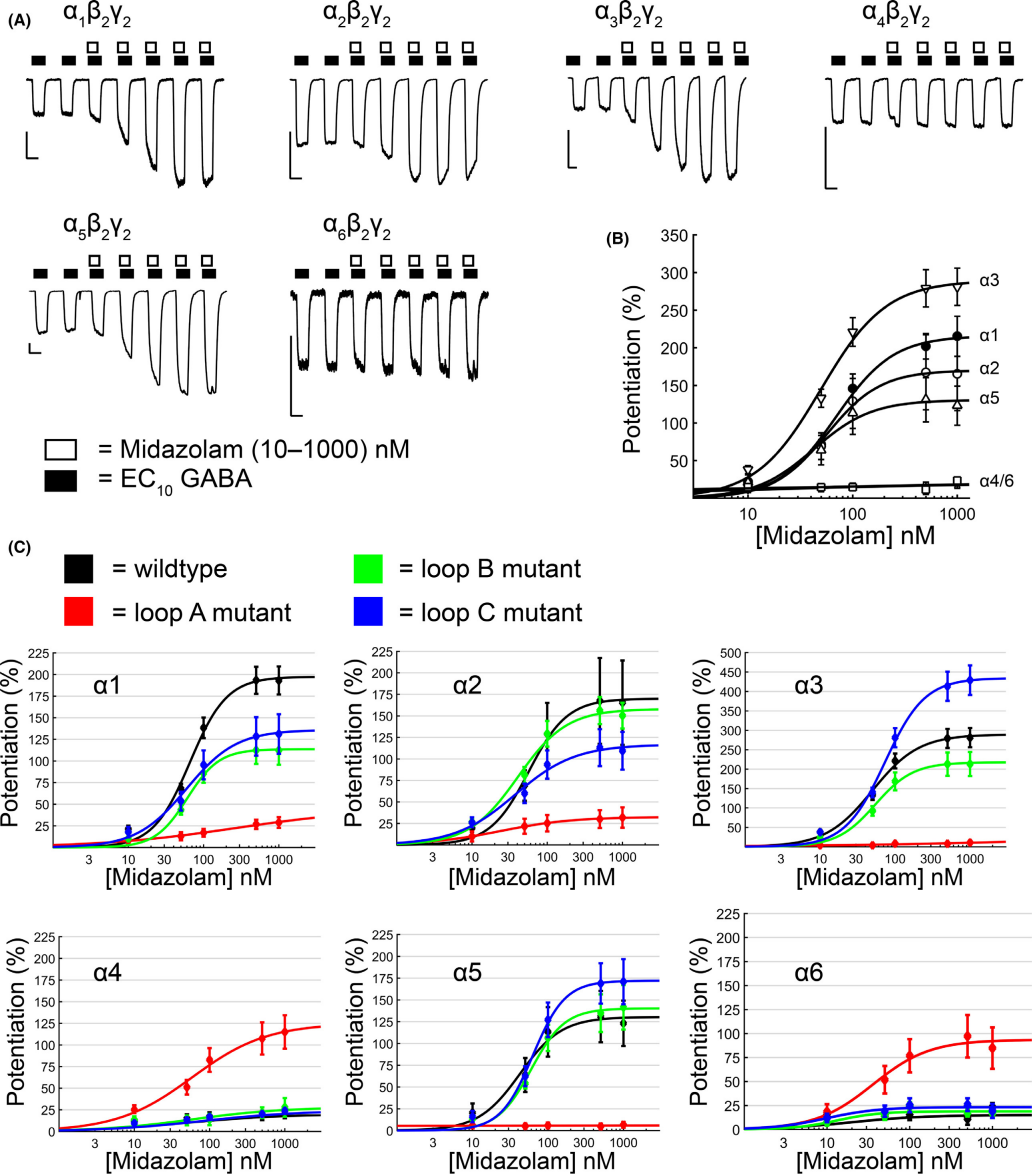
Mutations in loops A‐C of the α subunit alter the degree of midazolam potentiation measured at α_x_β_2_γ_2_ GABA_A_ receptors. (A) Example traces of midazolam (white boxes, 10‐1000 nM) potentiating EC_10_ GABA (black boxes) responses in wildtype α_x_β_2_γ_2_ receptors for α1‐6. Whole‐cell patch clamp recording was performed on HEK293T cells expressing α_x_β_2_γ_2_ receptors. Scale bars: 5 seconds, 500 pA. (B) Midazolam concentration‐response curves from wildtype α_x_β_2_γ_2_ GABA_A_ receptors for 10‐1000 nM midazolam. Potentiation (%) was measured as the percent of enhancement in peak current evoked by EC_10_ GABA. A 100% potentiation would be a doubling in current of the EC_10_ GABA control response. Legend: α1 (•), α2 (○), α3(▿), α4(*), α5(▵), α6(□). Points are mean ± SEM and where SEM is smaller than symbols, it is not visible. N = 9‐17 cells per group.(C) Midazolam concentration‐response curves from α_x_β_2_γ_2_ GABA_A_ receptors containing loop A‐C mutations and compared to their wildtype α_x_β_2_γ_2_ receptor counterparts. Potentiation (%) was measured as the percent of enhancement in peak current evoked by EC_10_ GABA. Each line represents a different receptor condition: wildtype (black), loop A (red), loop B (green), and loop C (blue). Loop A mutations are α1(H102R), α2(H101R), α3(H126R), α4(R100H), α5(H105R), and α6(R100H). Loop B mutations are α1(T163P), α2(T162P), α3(T187P), α4(P161T), α5(P166T), α6(P161T). Loop C mutations are α1(S206I), α2(S205I), α3(S230I), α4(I204S), α5(S209I), and α6(N204I). Points are mean ± SEM and where SEM is smaller than symbols, it is not visible. Sample sizes (cells per group) are: 9‐14 (α1), 7‐10 (α2), 6‐17 (α3), 6‐9 (α4), 7‐11 (α5), and 6‐8 (α6)

The loop A mutation substituted the highly conserved histidine residue for an arginine residue (FF**H**NG) in the α1, α2, α3, and α5 subunits. For the α4 and α6 subunits, the reverse arginine‐to‐histidine mutation was made. GABA concentration‐response assays revealed only modest changes in loop A mutant receptors (Figure 2, Table S1). The presence of the arginine right‐shifted the GABA concentration‐response curves for α5(H105R)‐ and α2(H101R)‐containing receptors. This caused a threefold increase in the GABA EC_50_ for α5(H105R) mutant receptors (α_5_(H105R)β_2_γ_2_ = 9.84 ± 3.29 μM (n = 10); α_5_β_2_γ_2 _= 3.18 ± 0.71 μM (n = 10), *P *=* *0.0093) and twofold increase in the GABA EC_50_ for the α2(H101R) mutant receptors (α_2_(H101R)β_2_γ_2_ = 16.25 ± 2.20 μM (*n* = 11); α_2_β_2_γ_2_ = 8.29 ± 0.78 μM (*n* = 40), *P *=* *0.0003). Midazolam assays showed that the α1(H102R), α2(H102R), α3(H126R), α5(H105R) mutations abolished the ability of receptors to respond to midazolam potentiation, and Hill fits could not be performed on this data (Table 1, see Table S2 for midazolam potentiation values). This is consistent with previous reports using diazepam.^11^ The α4(R100H) and α6(R100H) mutations conferred the ability to receptors to respond to midazolam potentiation (midazolam EC_50_: α_4_(R100H)β_2_γ_2_ = 73.99 ± 3.44 nM (n = 8) and α_6_(R100H)β_2_γ_2_ = 41.88 ± 6.02 nM (n = 7), Figure 3C). The wildtype α_4_β_2_γ_2_ and α_6_β_2_γ_2_ receptors showed no notable midazolam potentiation, and no meaningful Hill parameters could be estimated (Figure 3A‐B, see Table S2 for values). Confirming the role of this histidine in loop A with midazolam provided a reference for how altering a key structural residue in a conserved region of the benzodiazepine binding site can maximally alter the amplitude of midazolam potentiation of the α_x_β_2_γ_2_ GABA_A_ receptors.

**Table 1:** Midazolam Hill fit parameters for GABAA receptors with loop A‐C mutations in the benzodiazepine site of α1‐6. Data points were taken from midazolam concentration‐response relationships (10‐1000 nM) measured with whole‐cell patch clamp recording of HEK293T cells expressing αxβ2γ2 receptors

	Conditions	Wildtype	Loop A	Loop B	Loop C
		α_1_β_2_γ_2_	α1(H102R)	α1(T163P)	α1(S206I)
α_1_	Max potentiation (%)	203.0 ± 17.6	h.n.f.	127.7 ± 16.0**	135.8 ± 23.78**
	Hill coefficient	1.765 ± 0.165	h.n.f.	2.113 ± 0.154	1.568 ± 0.199
	EC_50_ (nM)	71.43 ± 5.80	h.n.f.	61.08 ± 3.72	59.77 ± 4.11
	N	7	11	11	6
		α_2_β_2_γ_2_	α2(H101R)	α2(T162P)	α2(S205I)
α_2_	Max potentiation (%)	169.6 ± 49.9	h.n.f.	158.2 ± 15.8	116.4 ± 23.0
	Hill coefficient	1.743 ± 0.133	h.n.f.	1.393 ± 0.073	1.362 ± 0.140
	EC_50_ (nM)	50.90 ± 5.05	h.n.f.	42.03 ± 2.86	41.65 ± 4.99
	N	7	7	6	8
		α_3_β_2_γ_2_	α3(H126R)	α3(T187P)	α3(S230I)
α_3_	Max potentiation (%)	267.8 ± 20.3	h.n.f.	219.6 ± 32.3	436.0 ± 39.4**
	Hill coefficient	1.503 ± 0.117	h.n.f.	1.963 ± 0.224**	1.655 ± 0.061
	EC_50_ (nM)	46.39 ± 7.44	h.n.f.	55.21 ± 2.91	73.56 ± 1.81**
	N	7	6	6	7
		α_4_β_2_γ_2_	α4(R100H)	α4(P161T)	α4(I204S)
α_4_	Max potentiation (%)	h.n.f.	113.8 ± 21.6	h.n.f.	h.n.f.
	Hill coefficient	h.n.f.	1.187 ± 0.150	h.n.f.	h.n.f.
	EC_50_ (nM)	h.n.f.	73.99 ± 3.44	h.n.f.	h.n.f.
	N	6	8	7	7
		α_5_β_2_γ_2_	α5(H105R)	α5(P166T)	α5(S209I)
α_5_	Max potentiation (%)	107.9 ± 20.3	h.n.f.	140.7 ± 23.7	175.1 ± 26.6
	Hill coefficient	2.632 ± 0.329	h.n.f.	3.661 ± 1.897	2.232 ± 0.334
	EC_50_ (nM)	52.84 ± 3.48	h.n.f.	53.28 ± 5.54	65.44 ± 2.76
	N	7	7	7	6
		α_6_β_2_γ_2_	α6(R100H)	α6(P161T)	α6(N204I)
α_6_	Max potentiation (%)	h.n.f.	93.27 ± 22.84	h.n.f.	h.n.f.
	Hill coefficient	h.n.f.	2.310 ± 0.56	h.n.f.	h.n.f.
	EC_50_ (nM)	h.n.f.	41.88 ± 6.02	h.n.f.	h.n.f.
	N	7	7	6	6

Fits were performed on each cell’s midazolam concentration response data. Midazolam concentration‐response relationships not described by a sigmoidal function (h.n.f. = Hill Not Fit) were not included in our analysis (eg: no response or a linear nonsaturating response). Significance was determined using one‐way ANOVA with Dunnett’s post hoc analysis for each α‐subunit and its mutations. **P < 0.05. Multiple comparisons were made relative to the wildtype αxβ2γ2 receptor. The wildtype receptors containing α4 and α6 subunits lacked sigmoidal relationships and no statistics could be run to compare parameters from α4(R100H) and α6(R100H) datasets. Values are mean ± S.E.M. from *N* number of cells.

### 3.2 Loop B mutations

The loop B mutations consisted of mutating a threonine‐to‐proline (GSYAY**T**R) in α1‐3, which we predicted would reduce the receptor's responsiveness to midazolam. The opposite mutation (proline‐to‐threonine) was made in the α4‐6 subunits. No significant (*P *<* *0.05) shifts in GABA apparent‐affinity were seen for any α1‐6 loop B mutations (Figure 2, Table S1). The only significant (*P *<* *0.05) changes in GABA activation were modest changes in the amplitude of the maximum whole‐cell current evoked by GABA for α1(T163P), α2(T162P), and α4(P161T) mutated receptors. The GABA EC_50_ values remained unaltered for these mutants (*P *>* *0.05, Table S1). The receptors containing threonine‐to‐proline mutations failed to abolish the receptors’ response to midazolam for α1(T163P), α2(T162P), and α3(T187P) mutants. The midazolam EC_50_ values of α1(T163P), α2(T162P), and α3(T187P) mutants remained unchanged relative to the wildtype receptors (*P *>* *0.05, Table 1). Only α_1_(T163P)β_2_γ_2_ receptors had a significantly lower maximum potentiation compared to wildtype α_1_β_2_γ_2_ receptors (α_1_(T163P)β_2_γ_2_: 133.8 ± 19.51%, n = 11; α_1_β_2_γ_2_: 203.0 ± 17.6%, n = 7, *P *=* *0.0092). The α5(P166T) mutation produced little change in midazolam potentiation, either maximum potentiation or midazolam EC_50_ (*P *>* *0.05, n = 7 per group). The presence of a threonine residue failed to confer midazolam responsiveness to α_4_(P161T)β_2_γ_2_ and α_6_(P161T)β_2_γ_2_ receptors (Figure 3C, potentiation values in Table S2). Overall, the presence of a proline in this location caused only subtle changes in both GABA‐activation and midazolam potentiation.

### 3.3 Loop C mutations

**Figure 4:**
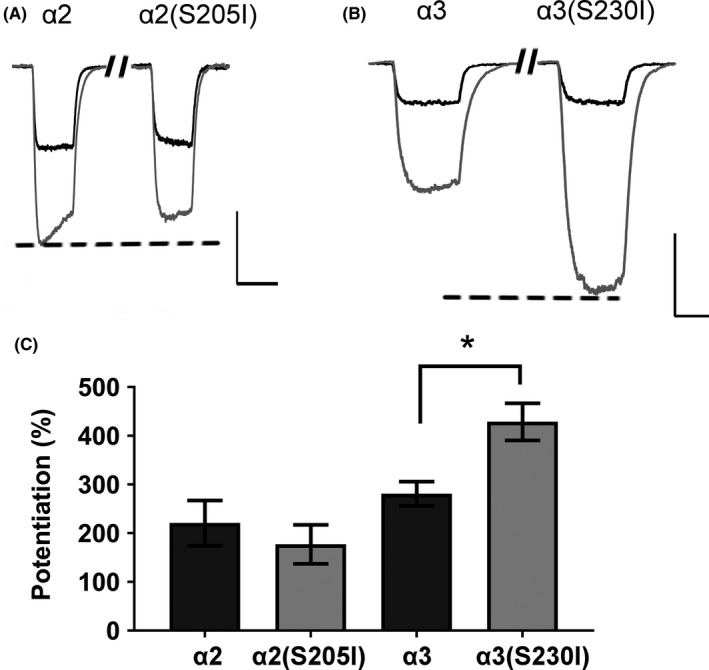
Although α2‐ and α3‐containing α_x_β_2_γ_2_ receptors had similar GABA apparent‐affinities (α2: EC_50_ = 8.29 ± 0.78 μM; α3: EC_50_ = 15.53 ± 2.55 μM), they showed different degrees of midazolam potentiation when the conserved serine in loop C of the α subunit was mutated to an isoleucine. (A‐B) Example traces of whole‐cell responses to EC_10_ GABA (black) and EC_10_ GABA + 1 μM midazolam (gray). (A) Example trace for α_2_β_2_γ_2_ and α_2_(S205I)β_2_γ_2_ receptors. Scale bar is 5 seconds, 500 pA. (B) Example trace for α_3_β_2_γ_2_ and α_3_(S230I)β_2_γ_2_ receptors. Scale bar is 5 seconds, 320 pA for α_3_β_2_γ_2_ and 5 seconds, 500 pA for α_3_(S230I)β_2_γ_2_ receptors. The dotted line marks the highest degree of midazolam potentiation for each example trace. (C) Quantifying the amplitude of maximum potentiation in the presence of 1 μM midazolam for α_2_β_2_γ_2_, α_2_(S205I)β_2_γ_2_, α_3_β_2_γ_2_ and α_3_(S230I)β_2_γ_2_ receptors. *P <* *0.05 significance was determined using a two‐way ANOVA with Sidak's post hoc analysis. Bars are mean ± SEM from n = 7‐8 cells per group

The loop C mutations (S**S**TGEYV) had little effect on GABA apparent‐affinity but more noticeable effects on the magnitude of the midazolam potentiation of α_x_β_2_γ_2_ GABA_A_ receptors. Five of the six loop C mutations failed to significantly (*P > *0.05) alter the receptor's apparent‐affinity for GABA, Table S1). The exception was α6(N204I) (EC_50_: α6(N204I)β_2_γ_2_ = 0.421 ± 0.061 μM (n = 14); α_6_β_2_γ_2_ = 0.703 ± 0.078 μM (n = 11), *P *=* *0.0001) (Figure 2). As predicted, the α1(S206I) mutation decreased the amplitude of the maximum potentiation by midazolam by approximately 33% (α_1_(S206I)β_2_γ_2_ = 135.8 ± 23.8% (n = 6); α_1_β_2_γ_2_ = 203.0 ± 17.6% (n = 7), *P *=* *0.0403). The α2(S205I) mutation reduced the maximum midazolam potentiation by approximately 31% (α_2_(S205I)β_2_γ_2_ = 116.4 ± 23.0%, n = 8) compared to wildtype receptors (α_2_β_2_γ_2_ = 169.6 ± 49.9%, n = 7), but this result was not significant (*P *=* *0.416). The α3(S230I) mutation had the largest alteration in midazolam potentiation (Figure 4). It enhanced the degree of maximum midazolam potentiation by approximately 63% (α_3_(S230I)β_2_γ_2_ = 436.0 ± 39.4% (n = 7); α_3_β_2_γ_2_ = 267.8 ± 20.3% (n = 7), *P *=* *0.0004), and it increased the midazolam EC_50_ by approximately 63% (α_3_(S230I)β_2_γ_2_ = 73.6 ± 1.8 nM; α_3_β_2_γ_2_ = 46.4 ± 7.4 nM, *P *=* *0.0014). Similarly, the α5(S209I) mutation increased the maximum degree of midazolam potentiation by approximately 63%, although this difference was not statistically significant (α_5_(S209I)β_2_γ_2_ = 175.1 ± 26.6% (n = 6); α_5_β_2_γ_2_ = 107.9 ± 20.3% (n = 7), *P *=* *0.1067). The α4(I204S) and α6(N204I) mutations failed to convey any notable midazolam potentiation to the receptors and no meaningful Hill parameters for midazolam concentration‐response curves could be estimated (Table 1). On the whole, loop C mutations showed that α_1_(S206I)β_2_γ_2_ and α_2_(S205I)β_2_γ_2_ receptors had a decreased maximal midazolam potentiation and the α_3_(S230I)β_2_γ_2_ and α_5_(S209I)β_2_γ_2_ receptors had an increased maximal potentiation.

Overall, mutating the threonine (loop B) and serine (loop C) residues failed to dramatically abolish the ability of α_x_β_2_γ_2_ GABA_A_ receptors to be modulated by midazolam, as has been established for the critical histidine in loop A. Mutations in loop C had a novel array of effects on midazolam efficacy, particularly for α3‐ and α5‐containing GABA_A_ receptors.

## 4 DISCUSSION

Midazolam is a benzodiazepine used to induce sedation and anesthesia.^23^ The therapeutically relevant range of midazolam measured from plasma is 75 ng/mL (207 nM, postoperative drowsiness) to 350 ng/mL (966 nM, anesthetized state).^26^, ^27^, ^28^ PAM benzodiazepines were initially thought to enhance the activity of GABA_A_ receptors by altering the GABA binding steps,^29^ but more recent models have focused on gating mechanisms.^30^, ^31^ The structural loops, loops A‐C within the α subunit, define half of the benzodiazepine site on GABA_A_ receptors. Understanding how different parts of the benzodiazepine site interact with modulators will help us better define the precise molecular mechanisms of these drugs.

In this study, we examined the role of the histidine in loop A, threonine in loop B, and serine in loop C within the α subunit and how these residues affected the allosteric potentiation of the GABA_A_ receptor by midazolam. The histidine‐to‐arginine loop A mutation provided an example of how a single residue mutation can dramatically alter the efficacy of midazolam potentiation. The loop B threonine and loop C serine are highly conserved across α subunits, except in α4 and α6 subunits which are generally insensitive to classic benzodiazepines.^22^, ^32^ We predicted that the presence of a proline in loop B and isoleucine in loop C would decrease the degree of potentiation of the α_x_β_2_γ_2_ GABA_A_ receptors by midazolam. Overall, the mutation of the conserved threonine‐to‐proline in loop B had subtle effects on midazolam potentiation. The serine‐to‐isoleucine mutation in loop C altered the efficacy of midazolam potentiation, especially for α3‐ and α5‐containing receptors.

Across the 18 mutations made in loops A‐C within the benzodiazepine site, only subtle changes were seen in GABA apparent‐affinity. Since the mutation was away from the GABA binding site, it is unlikely the mutations caused a structural rearrangement of the extracellular domain that affected the channel's activation. The α6(N204I) mutant increased the GABA's apparent‐affinity, but this was not sufficient to make the receptor any more responsive to midazolam than the wildtype α6‐containing receptors. On the whole, our results were consistent with mutations that had minimal effects on GABA's normal actions at the mutated receptor.

It is well‐established that the conserved histidine present in loop A (FF**H**NG) of the α subunit is important in determining the molecular 6, 11, 33 and behavioral 1 effects of benzodiazepines. This histidine is present in the α subunits sensitive to positive benzodiazepines, but in α4 and α6 isoforms that are insensitive, an arginine is present that sterically inhibits benzodiazepines from interacting properly with the receptor.^15^, ^22^ In our study, the histidine‐to‐arginine mutations in α1‐3 and α5 abolished midazolam potentiation, consistent with prior studies using diazepam.^11^ Conversely, mutating the conserved arginine‐to‐histidine in α4 and α6 conferred midazolam potentiation capabilities to α4(R100H)‐ and α6(R100H)‐containing α_x_β_2_γ_2_ receptors. These results provided an example of how a single residue mutation could dramatically alter the efficacy of midazolam potentiation across receptors containing α1‐6 isoforms.

One difficult aspect of measuring midazolam potentiation is choosing a drug application time that is sufficient to reach peak activation but avoids excessive desensitization and incomplete washout between applications. The 3 second midazolam exposure time used here, tried to balance these concerns while staying consistent across all receptor combinations used. Although incomplete peak responses at middle midazolam concentrations could result in a slightly under‐estimated EC_50_, the conclusions drawn here focus on the maximum potentiation measured at saturating midazolam concentrations as an estimation of drug efficacy.

In this study, the threonine (GSYAY**T**R, loop B) and serine (S**S**TGEYV, loop C) mutations had more subtle effects on midazolam potentiation than the α1(H102R) mutation. Our loop B results showed that only the α1(T163P) mutation decreased the maximum amplitude of midazolam potentiation as predicted. Of the other loop B mutations, α3(T187P) only slightly decreased the maximum potentiation, while α5(P166T) slightly increased it. Our results were consistent with the threonine in loop B conferring slightly higher midazolam efficacy to the receptor than the proline. This is consistent with a proline‐to‐threonine mutation in α5 and α6 that moderately increased zolpidem^21^ and diazepam^15^ binding affinities in previous studies.

The loop C mutation had more obvious changes in the efficacy of midazolam potentiation. The wildtype α1, α2, α3, and α5 subunits all contain the homologous Ser206 (human α1) that we predicted would reduce midazolam potentiation when mutated to an isoleucine. Surprisingly, the results did not follow the predicted pattern. In the α1(S206I) and α2(S205I) mutants, the isoleucine decreased midazolam's maximum potentiation by 31‐33%, but in α3(S230I) and α5(S209I), it increased midazolam's potentiation by approximately 63%. Only α3(S230I) significantly (*P *<* *0.05) altered midazolam's EC_50._ In the case of an allosteric modulator, an altered EC_50_ might be caused by changes in the modulator's ability to bind and interact with the receptor or the modulator's ability to alter GABA's binding and gating of the channel.^34^ As mentioned above, only modest changes in GABA apparent‐affinity were seen for loop C mutations, suggesting that any changes in midazolam potentiation were more likely caused by an altered midazolam‐receptor interaction and not global alterations in structure that transmitted to the GABA binding site.

Loop C is important for ligand binding because it has more mobility than the other loops 35 and may affect benzodiazepine ligand selectivity.^36^ Previous studies found that the α6(Asn204) and α4(Ile203) residues (both homologous to human α1(Ser206)) were important for distinguishing the binding of negative benzodiazepines.^19^ Ser206 also physically interacts with diazepam in α1, α2 and α5, suggesting a critical role in benzodiazepine action.^37^ However, a neighboring mutation, homologous to α1(T207C), specifically altered benzodiazepine efficacy and not binding.^12^ We propose that the homologous Ser206 in loop C may provide an important point of contact between the ligand and benzodiazepine site that affects the coupling of the benzodiazepine site to GABA activation, thereby affecting the benzodiazepine's efficacy. Because the effect of mutations in α3 and α5 were most dramatic, this serine may be more appropriately positioned in these subunits to alter midazolam's efficacy.

The α3 and α5 subunits have specific expression profiles in the brain that reflect their roles in cognitive‐ and limbic‐related pathways. The α3 subunit is expressed in the cortex, amygdala, olfactory bulb, and thalamic reticular nucleus, where α_3_β_2/3_γ_2_ receptors mediate phasic inhibition. The α5 subunit is most highly expressed in the pyramidal hippocampal cells but also in the cortex and hypothalamus.^4^, ^38^ The α_5_β_3_γ_2_ receptors contribute to tonic inhibition in the hippocampus 39 and have increasingly been studied for their role in cognition 40, 41 and anesthetic‐induced neurotoxicity.^42^

In our results, the greatest increase in midazolam's efficacy was seen with the α3(S230I) loop C mutation. The wildtype α3‐containing receptors were the most sensitive to modulation by midazolam with the lowest midazolam EC_50_ and highest maximum potentiation relative to the other α subunits. This is consistent with previous studies where diazepam and flunitrazepam potentiated α_3_β_1_γ_2_ receptors more than α_1_β_1_γ_2_ receptors.^43^, ^44^ Even with the higher wildtype levels of midazolam potentiation, the α3(S230I) loop C results were still notable. The α3(S230I) mutation in loop C dramatically increased the efficacy of midazolam potentiation compared to α2(S205I) (Figure 4) despite both α_2_β_3_γ_2_ and α_3_β_3_γ_2_ wildtype receptors having similar GABA apparent‐affinities (Figure S2). This novel finding underlines the importance of better understanding the differences in allosteric modulation of GABA_A_ receptors expressing α3 compared to other α subunits. For example, nonhypnotic drugs targeting the α2 and α3 subunits have been studied for their anxiolytic and analgesic effects.^41^, ^45^ However, creating ligands that distinguish these two subunits remains difficult, as shown when an “α3‐specific” PAM (SB‐205384) was found to potentiate α6‐containing GABA_A_ receptors even more strongly than α3.^46^ Another way to distinguish different GABA_A_ receptor subtypes is through the γ subunit. Although other γ subunits can form benzodiazepine‐sensitive receptors, the γ3 subunit is less prevalent (~14% of receptors),^47^ and the γ1 subunit notably reduces the benzodiazepine affinity of the receptor.^48^ The γ2 subunit is the major γ isoform expressed in native GABA_A_ receptors,^49^ and thus α_x_β_2_γ_2_ receptors provide a reasonable estimate of benzodiazepine efficacy in the brain. Based on our results, loop C might be a potential target for developing novel drugs that specifically modulate α3‐ and α5‐containing GABA_A_ receptors using PAMs targeting the allosteric benzodiazepine site.

## AUTHOR CONTRIBUTIONS

Participated in research design: O.A. Moody, A. Jenkins. Conducted experiments and data analysis: O.A. Moody. Wrote or contributed to the writing of the manuscript: O.A. Moody, A. Jenkins.

## DISCLOSURES

None declared.

## References

[1] Rudolph U , Crestani F , Mohler H . GABA_A_ receptor subtypes: dissecting their pharmacological functions. Trends Pharmacol Sci. 2001;22:188‐194. 11282419 10.1016/s0165-6147(00)01646-1

[2] Rudolph U , Mohler H . Analysis of GABA_A_ receptor function and dissection of the pharmacology of benzodiazepines and general anesthetics through mouse genetics. Annu Rev Pharmacol Toxicol. 2004;44:475‐498. 14744255 10.1146/annurev.pharmtox.44.101802.121429

[3] Fritschy JM , Mohler H . GABA_A_‐receptor heterogeneity in the adult rat brain: differential regional and cellular distribution of seven major subunits. J Comp Neurol. 1995;359:154‐194. 8557845 10.1002/cne.903590111

[4] Pirker S , Schwarzer C , Wieselthaler A , Sieghart W , Sperk G . GABA_A_ receptors: immunocytochemical distribution of 13 subunits in the adult rat brain. Neuroscience. 2000;101:815‐850. 11113332 10.1016/s0306-4522(00)00442-5

[5] Sieghart W . Structure and pharmacology of gamma‐aminobutyric acidA receptor subtypes. Pharmacol Rev. 1995;47:181‐234. 7568326

[6] Wieland HA , Lüddens H , Seeburg PH . A single histidine in GABA_A_ receptors is essential for benzodiazepine agonist binding. J Biol Chem. 1992;267:1426‐1429. 1346133

[7] Brejc K , van Dijk WJ , Klaassen RV , et al. Crystal structure of an ACh‐binding protein reveals the ligand‐binding domain of nicotinic receptors. Nature 2001;411:269‐276. 11357122 10.1038/35077011

[8] Kash TL , Trudell JR , Harrison NL . Structural elements involved in activation of the gamma‐aminobutyric acid type A (GABA_A_) receptor. Biochem Soc Trans. 2004;32:540‐546. 15157180 10.1042/BST0320540

[9] Cromer BA , Morton CJ , Parker MW . Anxiety over GABA_A_ receptor structure relieved by AChBP. Trends Biochem Sci. 2002;27:280‐287. 12069787 10.1016/s0968-0004(02)02092-3

[10] Miller PS , Aricescu AR . Crystal structure of a human GABA_A_ receptor. Nature. 2014;512:270‐275. 24909990 10.1038/nature13293 PMC4167603

[11] Benson JA , Low K , Keist R , Mohler H , Rudolph U . Pharmacology of recombinant gamma‐aminobutyric acidA receptors rendered diazepam‐insensitive by point‐mutated alpha‐subunits. FEBS Lett. 1998;431:400‐404. 9714551 10.1016/s0014-5793(98)00803-5

[12] Morlock EV , Czajkowski C . Different residues in the GABA_A_ receptor benzodiazepine binding pocket mediate benzodiazepine efficacy and binding. Mol Pharmacol. 2011;80:14‐22. 21447642 10.1124/mol.110.069542 PMC3127544

[13] Sancar F , Ericksen SS , Kucken AM , Teissere JA , Czajkowski C . Structural determinants for high‐affinity zolpidem binding to GABA_A_ receptors. Mol Pharmacol. 2007;71:38‐46. 17012619 10.1124/mol.106.029595

[14] Tan KR , Gonthier A , Baur R , Ernst M , Goeldner M , Sigel E . Proximity‐accelerated chemical coupling reaction in the benzodiazepine‐binding site of gamma‐aminobutyric acid type A receptors: superposition of different allosteric modulators. J Biol Chem. 2007;282:26316‐26325. 17626010 10.1074/jbc.M702153200

[15] Wieland HA , Luddens H . Four amino acid exchanges convert a diazepam‐insensitive, inverse agonist‐preferring GABA_A_ receptor into a diazepam‐preferring GABA_A_ receptor. J Med Chem. 1994;37:4576‐4580. 7799410 10.1021/jm00052a019

[16] Wieland M , Hartig JS . RNA quadruplex‐based modulation of gene expression. Chem Biol. 2007;14:757‐763. 17656312 10.1016/j.chembiol.2007.06.005

[17] Rudolph U , Crestani F , Benke D , et al. Benzodiazepine actions mediated by specific gamma‐aminobutyric acid(A) receptor subtypes. Nature. 1999;401:796‐800. 10548105 10.1038/44579

[18] Buhr A , Schaerer MT , Baur R , Sigel E . Residues at positions 206 and 209 of the alpha1 subunit of gamma‐aminobutyric AcidA receptors influence affinities for benzodiazepine binding site ligands. Mol Pharmacol. 1997;52:676‐682. 9380031 10.1124/mol.52.4.676

[19] Derry JM , Dunn SM , Davies M . Identification of a residue in the gamma‐aminobutyric acid type A receptor alpha subunit that differentially affects diazepam‐sensitive and ‐insensitive benzodiazepine site binding. J Neurochem. 2004;88:1431‐1438. 15009644 10.1046/j.1471-4159.2003.02264.x

[20] Hanson SM , Morlock EV , Satyshur KA , Czajkowski C . Structural requirements for eszopiclone and zolpidem binding to the gamma‐aminobutyric acid type‐A (GABA_A_) receptor are different. J Med Chem. 2008;51:7243‐7252. 18973287 10.1021/jm800889m PMC2645942

[21] Renard S , Olivier A , Granger P , et al. Structural elements of the gamma‐aminobutyric acid type A receptor conferring subtype selectivity for benzodiazepine site ligands. J Biol Chem. 1999;274:13370‐13374. 10224099 10.1074/jbc.274.19.13370

[22] Knoflach F , Benke D , Wang Y , et al. Pharmacological modulation of the diazepam‐insensitive recombinant gamma‐aminobutyric acidA receptors alpha 4 beta 2 gamma 2 and alpha 6 beta 2 gamma 2. Mol Pharmacol. 1996;50:1253‐1261. 8913357

[23] Olkkola KT , Ahonen J . Midazolam and Other Benzodiazepines In: Schüttler J, Schwilden H, eds. Modern Anesthetics. Handbook of Experimental Pharmacology, vol.182 Berlin: Springer; 2008. 10.1007/978-3-540-74806-9_16 18175099

[24] Trudell JR , Yue ME , Bertaccini EJ , Jenkins A , Harrison NL . Molecular modeling and mutagenesis reveals a tetradentate binding site for Zn2 + in GABA_(A)_ alphabeta receptors and provides a structural basis for the modulating effect of the gamma subunit. J Chem Inf Model. 2008;48:344‐349. 18197653 10.1021/ci700324a

[25] Williams CA , Bell SV , Jenkins A . A residue in loop 9 of the beta2‐subunit stabilizes the closed state of the GABA_A_ receptor. J Biol Chem. 2010;285:7281‐7287. 20007704 10.1074/jbc.M109.050294 PMC2844176

[26] Glass PS , Bloom M , Kearse L , Rosow C , Sebel P , Manberg P . Bispectral analysis measures sedation and memory effects of propofol, midazolam, isoflurane, and alfentanil in healthy volunteers. Anesthesiology. 1997;86:836‐847. 9105228 10.1097/00000542-199704000-00014

[27] Persson MP , Nilsson A , Hartvig P . Relation of sedation and amnesia to plasma concentrations of midazolam in surgical patients. Clin Pharmacol Ther. 1988;43:324‐331. 3126014 10.1038/clpt.1988.39

[28] Persson P , Nilsson A , Hartvig P , Tamsen A . Pharmacokinetics of midazolam in total i.v. anaesthesia. Br J Anaesth. 1987;59:548‐556. 3580236 10.1093/bja/59.5.548

[29] Skerritt JH , Johnston GA . Enhancement of GABA binding by benzodiazepines and related anxiolytics. Eur J Pharmacol. 1983;89:193‐198. 6135616 10.1016/0014-2999(83)90494-6

[30] Kristiansen U , Lambert JD . Benzodiazepine and barbiturate ligands modulate responses of cultured hippocampal neurones to the GABA_A_ receptor partial agonist, 4‐PIOL. Neuropharmacology. 1996;35:1181‐1191. 9014133 10.1016/s0028-3908(96)00070-6

[31] Rusch D , Forman SA . Classic benzodiazepines modulate the open‐close equilibrium in alpha1beta2gamma2L gamma‐aminobutyric acid type A receptors. Anesthesiology. 2005;102:783‐792. 15791108 10.1097/00000542-200504000-00014

[32] Wafford KA , Thompson SA , Thomas D , Sikela J , Wilcox AS , Whiting PJ . Functional characterization of human gamma‐aminobutyric acidA receptors containing the alpha 4 subunit. Mol Pharmacol. 1996;50:670‐678. 8794909

[33] Kleingoor C , Wieland HA , Korpi ER , Seeburg PH , Kettenmann H . Current potentiation by diazepam but not GABA sensitivity is determined by a single histidine residue. NeuroReport 1993;4:187‐190. 8384024 10.1097/00001756-199302000-00018

[34] Colquhoun D . Binding, gating, affinity and efficacy: the interpretation of structure‐activity relationships for agonists and of the effects of mutating receptors. Br J Pharmacol. 1998;125:924‐947. 9846630 10.1038/sj.bjp.0702164 PMC1565672

[35] Michalowski MA , Kraszewski S , Mozrzymas JW . Binding site opening by loop C shift and chloride ion‐pore interaction in the GABA_A_ receptor model. Phys Chem Chem Phys. 2017;19:13664‐13678. 28429004 10.1039/c7cp00582b

[36] Hanson SM , Czajkowski C . Structural mechanisms underlying benzodiazepine modulation of the GABA_(A)_ receptor. J Neurosci. 2008;28:3490‐3499. 18367615 10.1523/JNEUROSCI.5727-07.2008 PMC2410040

[37] Luscher BP , Baur R , Goeldner M , Sigel E . Influence of GABA_(A)_ receptor alpha subunit isoforms on the benzodiazepine binding site. PLoS ONE. 2012;7:e42101. 22848717 10.1371/journal.pone.0042101 PMC3407089

[38] Lee V , Maguire J . The impact of tonic GABA_A_ receptor‐mediated inhibition on neuronal excitability varies across brain region and cell type.Front Neural Circuits 2014;8:3. 24550784 10.3389/fncir.2014.00003 PMC3909947

[39] Farrant M , Nusser Z . Variations on an inhibitory theme: phasic and tonic activation of GABA_(A)_ receptors. Nat Rev Neurosci. 2005;6:215‐229. 15738957 10.1038/nrn1625

[40] Mohler H . The legacy of the benzodiazepine receptor: from flumazenil to enhancing cognition in Down syndrome and social interaction in autism. Adv Pharmacol. 2015;72:1‐36. 25600365 10.1016/bs.apha.2014.10.008

[41] Rudolph U , Knoflach F . Beyond classical benzodiazepines: novel therapeutic potential of GABA_A_ receptor subtypes. Nat Rev Drug Discov. 2011;10:685‐697. 21799515 10.1038/nrd3502 PMC3375401

[42] Zurek AA , Yu J , Wang DS , et al. Sustained increase in alpha5GABA_A_ receptor function impairs memory after anesthesia. J Clin Investig. 2014;124:5437‐5441. 25365226 10.1172/JCI76669 PMC4348961

[43] Puia G , Vicini S , Seeburg PH , Costa E . Influence of recombinant gamma‐aminobutyric acid‐A receptor subunit composition on the action of allosteric modulators of gamma‐aminobutyric acid‐gated Cl‐ currents. Mol Pharmacol. 1991;39:691‐696. 1646944

[44] Wafford KA , Whiting PJ , Kemp JA . Differences in affinity and efficacy of benzodiazepine receptor ligands at recombinant gamma‐aminobutyric acidA receptor subtypes. Mol Pharmacol. 1993;43:240‐244. 8381510

[45] Lewter LA , Fisher JL , Siemian JN , et al. Antinociceptive effects of a novel alpha2/alpha3‐subtype selective GABA_A_ receptor positive allosteric modulator. ACS Chem Neurosci. 2017;8:1305‐1312. 28150939 10.1021/acschemneuro.6b00447 PMC5686453

[46] Heidelberg LS , Warren JW , Fisher JL . SB‐205384 is a positive allosteric modulator of recombinant GABA_A_ receptors containing rat alpha3, alpha5, or alpha6 subunit subtypes coexpressed with beta3 and gamma2 subunits. J Pharmacol Exp Ther. 2013;347:235‐241. 23902941 10.1124/jpet.113.207324 PMC3781410

[47] Quirk K , Gillard NP , Ragan CI , Whiting PJ , McKernan RM . gamma‐Aminobutyric acid type A receptors in the rat brain can contain both gamma 2 and gamma 3 subunits, but gamma 1 does not exist in combination with another gamma subunit. Mol Pharmacol. 1994;45:1061‐1070. 8022401

[48] Ymer S , Draguhn A , Wisden W , et al. Structural and functional characterization of the gamma 1 subunit of GABA_A_/benzodiazepine receptors. EMBO J. 1990;9:3261‐3267. 2170110 10.1002/j.1460-2075.1990.tb07525.x PMC552059

[49] Benke D , Mertens S , Trzeciak A , Gillessen D , Mohler H . GABA_A_ receptors display association of gamma 2‐subunit with alpha 1‐ and beta 2/3‐subunits. J Biol Chem. 1991;266:4478‐4483. 1847922

