# Supplementary material for: The role of loops B and C in determining the potentiation of GABAA receptors by midazolam
Source: Pharmacol Res Perspect. 2018 Nov 13;6(6):e00433. doi: 10.1002/prp2.433 (PMC6234229; doi:10.1002/prp2.433)
Supplement: Supplementary file 1 [file PRP2-6-e00433-s001.pdf]

Supplementary Tables and Figures:

The role of loops B and C in determining the potentiation of $GABA_A$ receptors by midazolam

Olivia A. Moody[1] and Andrew Jenkins[2]

[1]Neuroscience Program, Graduate Division of Biological and Biomedical Sciences, Laney Graduate School, Emory University, Atlanta, Georgia, United States

[2]Departments of Anesthesiology & Pharmacology, Emory University, Atlanta, Georgia, United States

The Journal of Pharmacology and Experimental Therapeutics

Supplementary Tables: 2

Supplementary Figures: 1

Table S1. Hill parameters for GABA concentration-response assays

	Conditions:	Wildtype	Loop A	Loop B	Loop C
		**$\alpha_1\beta_2\gamma_2$**	**α1(H102R)**	**α1(T163P)**	**α1(S206I)**
α_1	**Max Current (pA)**	-4218 ± 601.6	-3175 ± 444.3	-2449 ± 376.1**	-5007 ± 470.7
	**Hill coefficient**	1.400 ± 0.102	1.255 ± 0.097	1.230 ± 0.049	1.444 ± 0.104
	**EC_{50} (μM)**	45.10 ± 7.75	60.72 ± 5.23	56.81 ± 11.40	44.41 ± 10.66
	**N# (cells)**	10	10	10	10
		**$\alpha_2\beta_2\gamma_2$**	**α2(H101R)**	**α2(T162P)**	**α2(S205I)**
α_2	**Max Current (pA)**	-3326 ± 356.7	-3186 ± 390.8	-1765 ± 246.0**	-7835 ± 763.5**
	**Hill coefficient**	1.581 ± 0.074	1.443 ± 0.092	1.494 ± 0.101	1.452 ± 0.151
	**EC_{50} (μM)**	8.29 ± 0.78	16.25 ± 2.20**	11.63 ± 1.85	9.60 ± 1.13
	**N# (cells)**	40	11	16	9
		**$\alpha_3\beta_2\gamma_2$**	**α3(H126R)**	**α3(T187P)**	**α3(S230I)**
α_3	**Max Current (pA)**	-2535 ± 210.5	-3597 ± 343.6**	-1993 ± 189.7	-1724 ± 286.1
	**Hill coefficient**	1.467 ± 0.065	1.519 ± 0.88	1.388 ± 0.155	1.796 ± 0.063**
	**EC_{50} (μM)**	15.53 ± 2.55	24.39 ± 4.57	16.39 ± 2.23	14.46 ± 1.171
	**N# (cells)**	16	16	12	11
		**$\alpha_4\beta_2\gamma_2$**	**α4(R100H)**	**α4(P161T)**	**α4(I204S)**
α_4	**Max Current (pA)**	-3039 ± 347.3	-3049 ± 378.6	-4487 ± 397.2**	-3424 ± 394.5
	**Hill coefficient**	1.113 ± 0.063	1.215 ± 0.079	1.392 ± 0.073**	1.180 ± 0.072
	**EC_{50} (μM)**	3.00 ± 0.53	3.58 ± 0.62	3.61 ± 0.46	3.41 ± 0.70
	**N# (cells)**	12	12	14	13
		**$\alpha_5\beta_2\gamma_2$**	**α5(H105R)**	**α5(P166T)**	**α5(S209I)**
α_5	**Max Current (pA)**	-5115 ± 315.9	-6799 ± 919.2	-4543 ± 553.0	-6073 ± 742.0
	**Hill coefficient**	1.547 ± 0.123	1.269 ± 0.084	1.420 ± 0.064	1.434 ± 0.059
	**EC_{50} (μM)**	3.18 ± 0.71	9.84 ± 3.29**	1.94 ± 0.29	1.09 ± 0.57

	**N# (cells)**	10	10	12	10
		**$\alpha_6\beta_2\gamma_2$**	**α6(R100H)**	**α6(P161T)**	**α6(N204I)**
α_6	**Max Current (pA)**	-3276 ± 578.1	-2902 ± 349.5	-3540 ± 290.2	-3549 ± 408.6
	**Hill coefficient**	1.405 ± 0.074	1.277 ± 0.079	1.323 ± 0.063	1.260 ± 0.056
	**EC_{50} (μM)**	0.703 ± 0.078	0.570 ± 0.110	0.575 ± 0.056	0.421 ± 0.061**
	**N# (cells)**	11	7	15	14

Table S1. Hill parameters estimated from GABA concentration-response relationships for benzodiazepine site mutations in loops A-C of the α subunit. Residues of interest were the histidine/arginine (loop A), the threonine/proline (loop B), and the serine/isoleucine (loop C). Whole-cell patch clamp recording was performed on HEK293T cells expressing $\alpha_x\beta_2\gamma_2$ receptors. Significance was determined using one-way ANOVA tests (α = 0.05) for each α subunit and its loop A-C mutations (4 receptor conditions). Where significance was found, a Dunnett's post-hoc analysis for multiple comparisons was performed using the wildtype receptors as the control group. Asterisks denote *p*<0.05 significance. Sample sizes were from N cells. Values are mean ± S.E.M

Table S2. Midazolam potentiation measurements for midazolam assays

		**Midazolam Potentiation Values**			
	**Conditions:**	**Wildtype**	**Loop A**	**Loop B**	**Loop C**
	**[MDZ] nM**	**$\alpha_1\beta_2\gamma_2$**	**α1(H102R)**	**α1(T163P)**	**α1(S206I)**
α_1	**10**	22.06 ± 3.46	8.10 ± 3.48	14.19 ± 4.15	13.37 ± 5.70
	**50**	71.67 ± 6.40	13.07 ± 4.69	49.96 ± 8.44	44.00 ± 10.96
	**100**	145.94 ± 13.37	16.99 ± 4.42	96.68 ± 15.00	80.14 ± 17.04
	**500**	201.94 ± 16.80	26.78 ± 5.31	124.27 ± 18.98	107.07 ± 22.95
	**1000**	215.35 ± 26.71	28.87 ± 6.18	123.00 ± 18.99	108.21 ± 24.13
	**N**	7 (13)	11 (15)	11 (18)	6 (11)
	**[MDZ] nM**	**$\alpha_2\beta_2\gamma_2$**	**α2(H101R)**	**α2(T162P)**	**α2(S205I)**
α_2	**10**	19.47 ± 4.19	9.85 ± 5.77	26.45 ± 4.06	21.06 ± 6.24
	**50**	69.08 ± 17.73	21.72 ± 8.75	81.24 ± 9.43	54.26 ± 10.25
	**100**	128.98 ± 36.14	25.17 ± 9.58	129.24 ± 14.94	86.20 ± 14.74
	**500**	167.38 ± 49.89	30.08 ± 10.31	156.21 ± 15.70	102.63 ± 19.49
	**1000**	165.35 ± 48.98	31.81 ± 11.79	150.36 ± 15.00	97.62 ± 20.12
	**N**	7 (10)	7 (11)	6 (9)	8 (13)
	**[MDZ] nM**	**$\alpha_3\beta_2\gamma_2$**	**α3(H126R)**	**α3(T187P)**	**α3(S230I)**
α_3	**10**	37.98 ± 5.40	3.71 ± 1.31	23.71 ± 6.37	37.59 ± 4.12
	**50**	132.92 ± 12.09	3.63 ± 2.06	92.47 ± 12.51	139.86 ± 12.63
	**100**	221.10 ± 19.12	8.25 ± 1.64	168.56 ± 23.08	280.95 ± 24.73
	**500**	279.18 ± 24.71	7.92 ± 2.84	212.97 ± 30.06	413.27 ±37.70
	**1000**	281.17 ± 24.73	10.78 ± 2.42	213.30 ± 30.99	428.92 ± 38.09
	**N**	7 (17)	6 (9)	6 (11)	7 (14)
	**[MDZ] nM**	**$\alpha_4\beta_2\gamma_2$**	**α4(R100H)**	**α4(P161T)**	**α4(I204S)**
α_4	**10**	10.98 ± 3.61	26.12 ± 4.95	13.22 ± 3.76	9.47 ± 1.93
	**50**	13.30 ± 4.65	54.65 ± 8.66	10.31 ± 5.64	13.23 ± 1.68
	**100**	15.15 ± 4.85	87.48 ± 13.68	14.28 ± 5.87	16.59 ± 1.59
	**500**	15.98 ± 5.42	117.13 ± 19.82	21.94 ± 5.61	20.16 ± 2.01
	**1000**	17.89 ± 4.82	125.77 ± 21.01	26.43 ± 9.72	23.95 ± 2.33
	**N**	6 (9)	8 (15)	7 (10)	7 (10)

	[MDZ] nM	$\alpha_5\beta_2\gamma_2$	α5(H105R)	α5(P166T)	α5(S209I)
α_5	**10**	20.47 ± 10.62	8.59 ± 4.39	13.32 ± 1.68	15.95 ± 6.58
	**50**	63.82 ± 19.54	7.73 ± 4.67	54.02 ± 5.66	62.43 ± 11.51
	**100**	113.29 ± 28.44	8.00 ± 4.85	106.37 ± 15.09	127.16 ± 19.87
	**500**	130.90 ± 29.52	5.65 ± 3.99	134.76 ± 21.59	168.94 ± 23.17
	**1000**	123.12 ± 26.08	8.17 ± 4.66	141.51 ± 25.59	170.92 ± 25.92
	**N**	7 (11)	7 (11)	7 (11)	6 (9)
	**[MDZ] nM**	$\alpha_6\beta_2\gamma_2$	**α6(R100H)**	**α6(P161T)**	**α6(N204I)**
α_6	**10**	14.66 ± 3.92	14.39 ± 8.03	7.52 ± 3.56	11.98 ± 4.42
	**50**	14.55 ± 3.86	45.91 ± 12.95	13.89 ± 3.99	19.07 ± 5.84
	**100**	14.58 ± 4.87	71.31 ± 17.31	24.46 ± 3.86	25.21 ± 7.32
	**500**	11.15 ± 6.25	85.96 ± 19.92	17.25 ± 3.52	25.85 ± 6.62
	**1000**	23.34 ± 4.23	79.13 ± 19.87	16.98 ± 4.08	19.31 ± 7.32
	**N**	7 (17)	7 (10)	6 (10)	6 (11)

Table S2. Midazolam potentiation (%) values measured from $GABA_A$ receptors containing mutations in the benzodiazepine site. Mutations in loops A-C were made across the α1-6 subunits. Potentiation was calculated as the percent of enhancement in peak current compared to EC_{10} GABA-evoked responses. Midazolam (MDZ) concentrations were from 10-1000 nM. Data was collected using whole-cell patch clamp recording of HEK293T cells expressing $\alpha_x\beta_2\gamma_2$ receptors. Sample sizes were from N cells with the total number midazolam assays run in parentheses. Values are mean ± S.E.M.

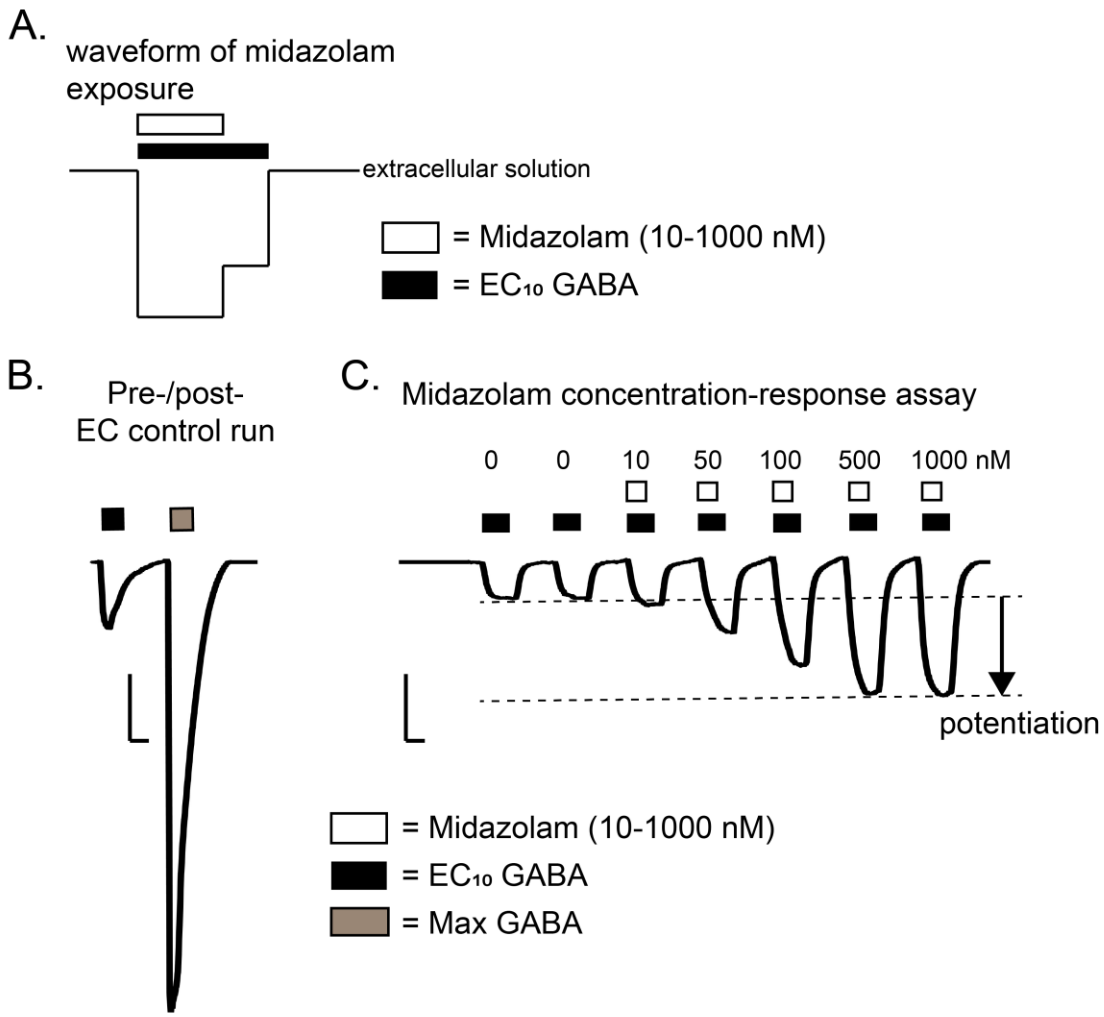

Supplementary Figure S1. Example of whole-cell patch clamp recording protocols used for midazolam concentration-response assays. **A)** Example waveform of the GABA + midazolam drug exposure used for midazolam concentration-response assays. GABA and midazolam were co-applied for 3 seconds and then GABA was applied alone for 2 sec before washout in extracellular solution. **B)** Example trace of the “Pre/post EC run” that was used to ensure that the chosen EC_{10} GABA concentration gave a 10% of maximum current response for the cell patched. This consisted of one 3 sec exposure to EC_{10} GABA then a 3 sec exposure to a saturating GABA concentration (max GABA). This protocol was run before and after each midazolam concentration-response to ensure consistent EC_{10} GABA responses and complete washout of midazolam afterwards. Scale bar: 5 sec, 500 pA. **C)** Example trace of midazolam concentration-response assay (10-1000 nM) for α_3(S230I)$\beta_2\gamma_2$ receptors. “Potentiation” is marked as arrow between dotted lines. Drug exposures were 5 seconds total before washout.

See *in vitro electrophysiology* in Methods for further details. *EC* = effective concentration. Scale bar: 5 sec, 500 pA.

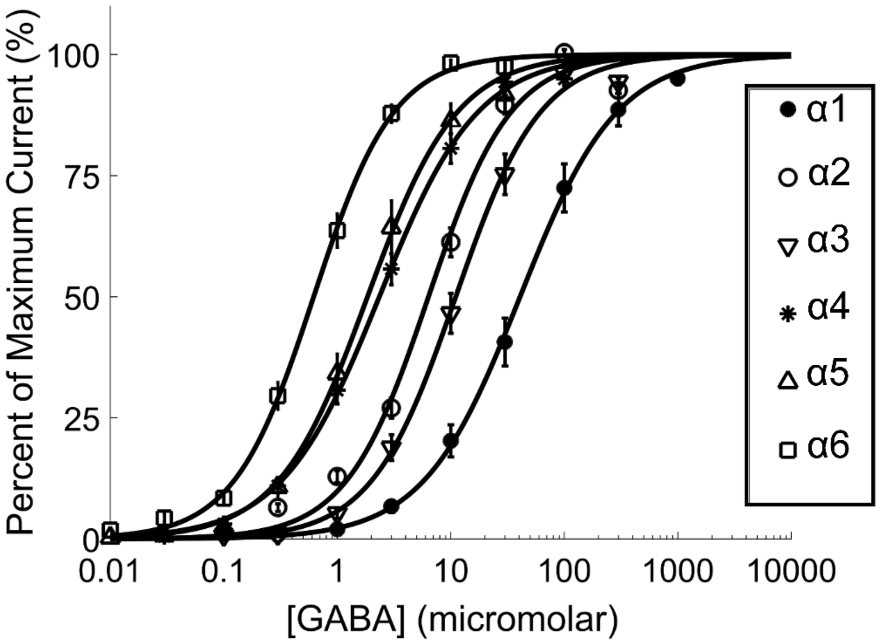

Supplementary Figure S2. GABA concentration-response curves for wildtype $\alpha_x\beta_2\gamma_2$ $GABA_A$ receptors. Data was measured using whole-cell patch clamp recording of HEK293T cells expressing $\alpha_x\beta_2\gamma_2$ receptors. Symbols are: α1 (●), α2 (○), α3(▽), α4(✱), α5(△), α6(□). GABA concentrations were: α1 = 0.3-1000 μM, α2, α3 = 0.01-300 μM, α4 = 0.03-100 μM and α5, α6 = 0.01-30 μM. Average GABA EC_{50} values were: 45.10 ± 7.75 μM (α1), 8.29 ± 0.78 μM (α2), 15.53 ± 2.55 μM (α3), 3.00 ± 0.53 μM (α4), 3.18 ± 0.71 μM (α5) and 0.703 ± 0.078 μM (α6). Lines are fit by eye and have no theoretical value. Points are mean ± S.E.M. and where S.E.M. is smaller than symbols, it is not visible. N = 10-40 cells per group.
